# Lung Magnetic Resonance Imaging with Diffusion Weighted Imaging Provides Regional Structural as well as Functional Information Without Radiation Exposure in Primary Antibody Deficiencies

**DOI:** 10.1007/s10875-015-0172-2

**Published:** 2015-06-12

**Authors:** Cinzia Milito, Federica Pulvirenti, Goffredo Serra, Michele Valente, Anna Maria Pesce, Guido Granata, Carlo Catalano, Francesco Fraioli, Isabella Quinti

**Affiliations:** Department of Molecular Medicine, Sapienza University of Rome, Viale dell’Università 37, Rome, Italy; Department of Radiological, Oncological and Pathological Sciences, Sapienza University of Rome, Rome, Italy; Department of Nuclear Medicine, University College London Hospital, London, UK; Division of Imaging, University College London Hospital, London, UK

**Keywords:** Primary antibody deficiencies, lung abnormalities, magnetic resonance, diffusion weighted imaging, inflammation, GLILD

## Abstract

**Purpose:**

Primary antibody deficiency patients suffer from infectious and non-infectious pulmonary complications leading over time to chronic lung disease. The complexity of this pulmonary involvement poses significant challenge in differential diagnosis in patients with long life disease and increased radio sensitivity. We planned to verify the utility of chest Magnetic Resolution Imaging with Diffusion-Weighted Imaging as a radiation free technique.

**Methods:**

Prospective evaluation of 18 patients with Common Variable Immunodeficiency and X-linked Agammaglobulinemia. On the same day, patients underwent Magnetic Resonance Imaging with Diffusion Weighted Imaging sequences, High Resolution Computerized Tomography and Pulmonary Function Tests, including diffusing capacity factor for carbon monoxide. Images were scored using a modified version of the Bhalla scoring system.

**Results:**

Magnetic Resonance Imaging was non-inferior to High Resolution Computerized Tomography in the capacity to identify bronchial and parenchymal abnormalities. HRCT had a higher capacity to identify peripheral airways abnormalities, defined as an involvement of bronchial generation up to the fifth and distal (scores 2–3). Bronchial scores negatively related to pulmonary function tests. One third of consolidations and nodules had Diffusion Weighted Imaging restrictions associated with systemic granulomatous disease and systemic lymphadenopathy. Lung Magnetic Resolution Imaging detected an improvement of bronchial and parenchymal abnormalities, in recently diagnosed patients soon after starting Ig replacement.

**Conclusions:**

Magnetic Resonance Imaging with Diffusion Weighted Imaging was a reliable technique to detect lung alterations in patients with Primary Antibody Deficiencies.

**Electronic supplementary material:**

The online version of this article (doi:10.1007/s10875-015-0172-2) contains supplementary material, which is available to authorized users.

## Introduction

Patients with X-linked Agammaglobulinemia (XLA) and Common Variable Immunodeficiency (CVID) suffer from recurrent respiratory infections and non-infectious pulmonary diseases possibly leading, over time, to permanent lung damage [1–4]. Common lung alterations are bronchiectasis, bronchial wall thickening, nodules and parenchymal consolidations [5, 6]. Moreover, patients with primary antibody deficiencies (PAD) are affected also by a group of lung diseases, denominated granulomatous-lymphocytic interstitial lung disease (GLILD) [7–11] characterized by granulomatous and pulmonary lymphoid hyperplasia, histological patterns consisting of lymphocytic interstitial pneumonia (LIP), follicular bronchiolitis, and lymphoid hyperplasia [12]. The complexity of this pulmonary involvement poses a significant challenge in differential diagnosis [13]. Physical examination, laboratory analysis and Pulmonary Function Tests (PFTs) are routinely used in the work-up of PAD lung disease, but High Resolution Computed Tomography (HRCT) is often required to establish a definite diagnosis. HRCT scan is the gold standard for the assessment of lung diseases. Because of the long life course of PAD, patients undergo a considerable number of radiological examinations during their life. As an increased radio sensitivity has been described [14–19], clinicians should consider a risk-benefit assessment when ordering a CT scan. We have already shown that Magnetic Resonance Imaging (MRI), a radiation-free technique was complementary or alternative to HRCT scan [20] to detect lung alterations. However, chest HRCT scan and morphological MRI sequences did not give information on the presence of *foci* of active inflammation and often invasive methods as lung biopsy or bronchoalveolar lavage are necessary [13]. Functional MRI with diffusion-weighted imaging (DWI) sequences might represent a promising tool for the identification of areas of active inflammation/sub clinical infections in lung parenchyma [21]. DWI signal derives from the free motion of water molecules in tissues. The interaction with an increased number of cells or macromolecules leads to a restriction of this motion, with higher DWI signal intensity. DWI has been validated as diagnostic tool in various inflammatory diseases in the abdomen, pelvis and brain [22–24].

In lung imaging, DWI has been applied in the differential diagnosis of lung consolidations and neoplasms, based on the fact that the signal intensity is higher in viable tumour tissues than in less densely packed tissues, such as tumour necrosis or benign consolidations [25–27].

Following these considerations, we have introduced DWI in our diagnostic approach for PAD patients. The objective of this study was to evaluate if chest MRI with DWI may represent a feasible radiation free option in the evaluation of PAD lung disease.

## Methods

### Study Design

We set out a prospective single center study, enrolling 18 consecutive patients aged >18 years with an established diagnosis of PAD [28] and recurrent respiratory infection. On the same day, patients underwent chest MRI without contrast medium and including DWI sequences and Pulmonary Function Tests (PFTs), including diffusing capacity factor for carbon monoxide (D_LCO_). To validate the MRI score, a chest HRCT scan was performed. Examinations were carried out far from clinical evidence of acute pulmonary infection. Data on clinical manifestation and immunoglobulin replacement were collected by medical records. Immuno-phenotyping of peripheral blood mononuclear cells was also performed. The institutional review board approved the study and a signed informed consent was obtained from all participants.

### HRCT Study

HRCT images were acquired with a 64-detector row spiral CT scanner (Volume Sensation Cardiac; Siemens), without injection of IV contrast agent, from the lung apices to the upper abdomen in a single breath-hold at the end of full inspiration. The parameters used for acquisition were as follows: 100 kV, CAREdose (Siemens) with quality reference set at 100 mAs; collimation, 64 × 0.6 mm; gantry rotation time, 0.33 s; scan time, 6 s; reconstruction kernel, B30 (for mediastinal evaluation) and B60 (for lung parenchyma evaluation).

### MRI Study

MRI images without contrast medium were obtained with a 1.5 Tesla scanner (Magnetom Avanto, Siemens, Enlargen, Germany) using a respiratory triggered DP weighted BLADE sequence (TR:2000 ms; TE = 27 ms; FOV 400 mm; flip angle: 150°; slice thickness 5 mm) acquired on an axial plane. DWI sequence (TR: 5632 ms, 12; TE: 83 ms; flip angle: 90°; FOV 400 mm slice thickness 5 mm; b0-b800 s/mm2) on an axial plane was included in the acquisition protocol. A combination of two surface coils covering the whole thorax and the spine was used.

### Image Analysis

MRI and CT scan studies were scored adopting the Bhalla score [20, 29] in a random order by two chest radiologists, blinded to patients’ identities and clinical information. Readers evaluated each examination independently and separately and reached an agreement for the controversial lesions. In order to reduce recall bias effects from the remembering of MRI findings, HRCT scans were evaluated 4 weeks after the analysis of the MR images. DWI abnormalities (hot spots) were identified as areas showing equal or higher signal intensity compared to the spinal cord at the highest b value [26]. An eyeballing comparison was performed between DWI and morphological-MRI in order to identify the morphological substrate of the diffusion abnormalities observed in consolidations or nodules.

### Bhalla Score

Classical Bhalla score evaluated severity and extent of bronchial and parenchymal parameters [29]. Bronchial parameters were: a) bronchiectasis severity, b) extent of bronchiectasis (number of lung segments), c) peribronchial thickening, d) extent of mucus plugs (number of lung segments), e) generation of bronchial division involved (bronchiectasis/plug). Parenchymal parameters were: a) abscesses or sacculations (number of lung segments), b) numbers of bullae, c) emphysema (number of lung segments), d) collapse/consolidations. Two extra parenchymal categories were introduced for the assessment of nodules: nodule number (score 0: no nodules detected; score 1: < 5 nodules detected; score 2: > 5 nodules detected) and size: (score 1: < 1 cm; score 2: > 1 cm). The cut-off diameter discriminating between nodules and consolidations was up to 30 mm. Total bronchial score (min 0; max 15) was defined as the sum of each bronchial parameter score; total parenchymal score (min 0; max 14) was defined as the sum of each parenchymal parameter score; total Bhalla score was defined as the sum of bronchial and parenchymal scores.

### PFTs

The PFTs were performed at the Pulmonary Function Unit. We analysed the following data sets: 1) forced vital capacity (FVC), 2) forced expiratory volume in one second (FEV1); 3) forced expiratory flow between 25 and 75 % (FEF25–75); 4) corrected D_LCO_.

### Flow Cytometry Analysis

Peripheral blood mononuclear cells were obtained by density-gradient centrifugation. Immunophenotyping was performed with the combination of 4 fluorochrome-labeled monoclonal antibodies (BD Biosciences). The following B-cell populations were analysed: classical naïve (CD19^+^CD27^−^CD21^+^CD38^+^), switched memory (CD19^+^CD27^+^CD21^+^IgM^−^), IgM memory (CD19^+^CD27^+^IgM^+^IgD^+^), transitional (CD19^+^IgM^++^CD38^++^) and CD21^low^ (CD19^+^CD21^−/low^CD38^−^). Dead cells were excluded from analysis by side/forward scatter gating. FACS analyses were performed on a FACSCalibur instrument (BD Biosciences) using the CellQuest (BD) and FlowJo (Tree Star) software.

### Statistical Analysis

Statistical analysis was performed by using a dedicated software (StatView). Descriptive data were presented as median and interquartile range (IQR), as indicated. Comparison of clinical and radiological changes between groups, was performed by Mann-Whitney test. Comparison of categorical and non-continuous variables between groups was performed by chi-square test. Comparison of MRI and HRTC scores was performed by chi square test to assess MRI non-inferiority. Comparison between single PFTs parameter and MRI score was assessed by simple linear regression analysis. A p value of <0.05 was taken as the threshold of statistical significance.

Inter-observer agreement was evaluated comparing the HRCT and MRI total scores (TOT) calculated by the 2 readers; intra-observer variability was evaluated comparing the two different readings of the same HRCT and MRI datasets performed by the most experienced radiologist after an interval of at least 4 weeks. The intra- and inter-observer agreement for scoring values were analysed by the use of Intraclass Correlation Coefficient (ICC).

## Results

### Patient Population

Eighteen (13 males, 5 females) PAD patients were enrolled in the study: 16 patients had a diagnosis of CVID and two patients of XLA. The median age at the study time was 39.5 years (IQR 30.5–55). At the enrolment, 14 patients received Ig replacement therapy administered intravenously (IVIG) and two patients subcutaneously (SCIG); 2 patients (n.4 and 9) were newly diagnosed CVID and they started the IVIG substitutive treatment at the time of the study, reaching a plateau of trough IgG level 3 months thereafter. PAD-associated clinical conditions were: autoimmune disease (8/18 patients), chronic lymphadenopathy (10/18 patients), splenomegaly (11/18 patients), systemic granulomatous disease (seen by histology – 4/18) and chronic obstructive lung disease (COPD) (7/18 patients). Relevant clinical and immunologic data were summarized in Supplementary Table 1. All patients with COPD diagnosis were on treatment with long-acting inhaled therapy (beta-agonists and steroids and/or anticholinergics). None of our patients were on treatment with systemic steroids and/or immunosuppressive drugs. Overall, on PFTs, FEV1 was 68 % (IQR 45–85 %), FVC: 70.5 % (IQR 53–81 %), FEV1/FVC: 97 % (IQR 73–107 %), PEF: 64 % (IQR 43–79 %), FEF 25–75: 71 % (IQR 32–98 %); DLCO (corrected): 91.6 % (IQR 78.6–96.7 %).

### CT and MRI: Image Acquisitions

Room time for CT scan was 4.1 min (IQR 3.9–5.2) and the dose length product was 104 mGy × cm (IQR: 90–113); effective dose was 1.3 mSv (IQR: 0.9–1.8). MRI room time was 17 min (IQR 12–20). Intra-observer agreement was calculated to assess reproducibility of the newly developed DWI scoring system. A good inter-observer agreement was found for both bronchial and parenchymal MRI scores (ICC for Total Score: 0903 and 0942 respectively). A good inter-observer agreement was observed for DWI scores (ICC for Total DWI score: 0975). A good inter-observer agreement between the two readers was found in the scoring evaluation of HRCT studies (ICC for Total Score: 0971).

### Lung Abnormalities by MRI

Mean total score recorded by MRI was 8 (IQR 5–11). Bronchial abnormalities and parenchymal abnormalities were evident in 78 % and in 66.7 % patients, respectively. Individual bronchial and parenchymal MRI scores were shown in Supplementary Table 2. Mean total bronchial MRI score was 6 (IQR 3.25–9). Bronchiectasis, mucus plugging, peribronchial thickening were detected in 77.8, 55.5 and 66.7 % of patients, respectively. As expected, PAD patients with COPD had higher total MRI bronchial scores (9, IQR 7–11) in comparison to patients without COPD (5, IQR 1.5–6) (*p* = 0.033). Total MRI parenchymal scores were 2 (IQR 0–6). None of the patients had bullae or abscesses; 16.7 % patients had emphysema. Consolidations and nodules were recorded in 50 and 55.5 % patients, respectively. The cumulative number of consolidations was 41 and of nodules was 40.

### HRCT and MRI Comparison in Lung Abnormalities Evaluation

Bronchial abnormalities were the most common lung alterations recorded in PAD patients by HRCT scan (89 %). We observed high levels of concordance between HRCT and MRI in scoring bronchiectasis severity (*p* = 0.0004), extent of bronchiectasis (*p* < 0.0001) and mucus plugging (*p* < 0.0001). A lower but still significant level of concordance was found between HRCT and MRI in the capacity to score the bronchial division involvement (*p* = 0.02), in that HRCT had a higher capacity (*p* = 0.02) to identify peripheral airways abnormalities, defined as an involvement of bronchial generation up to the fifth and distal (scores 2–3) detected in 83 and 61 % of patients, respectively.

**Fig. 1:**
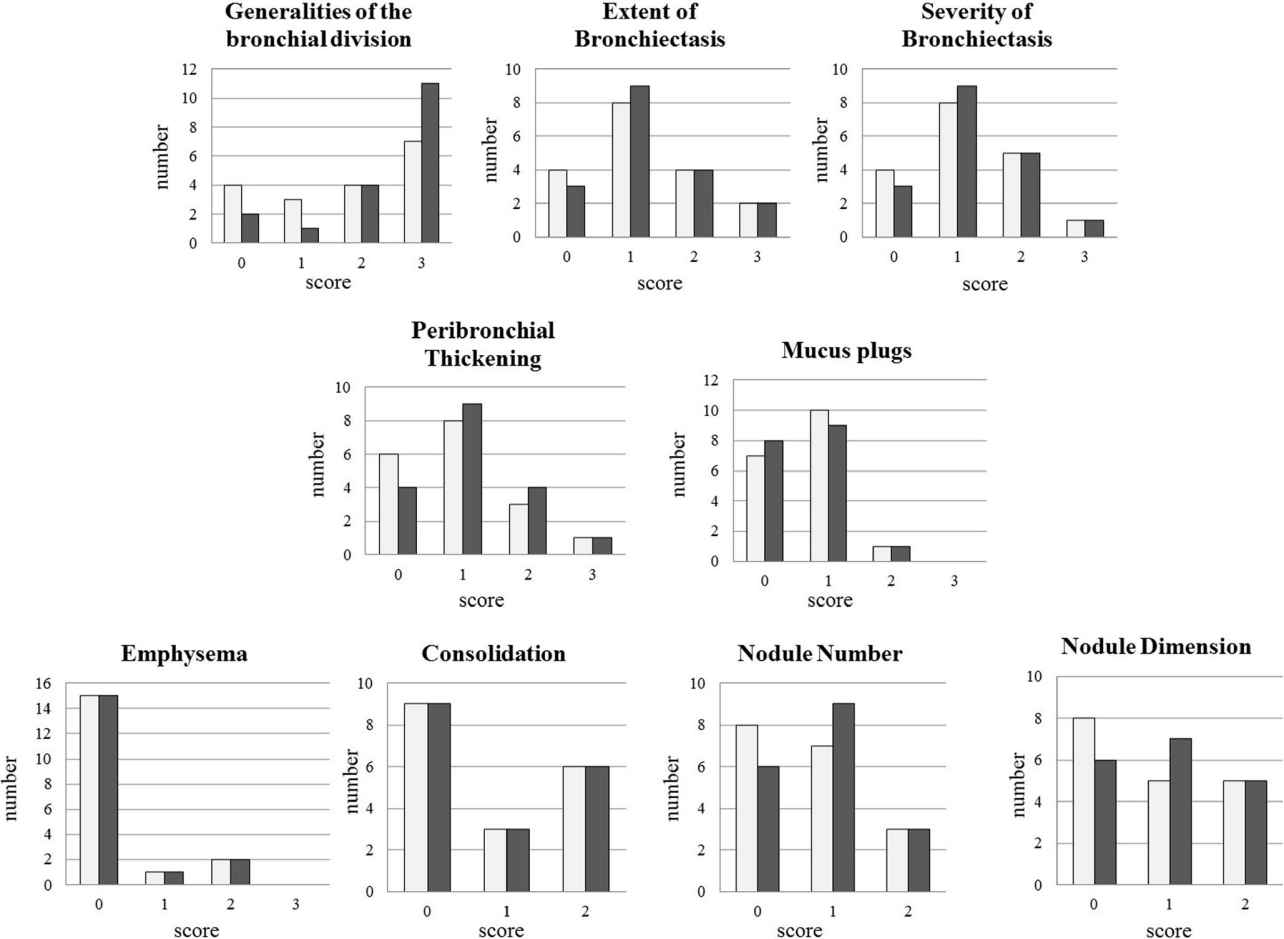
HRCT and MRI score comparison **a** Number of patients with primary antibody deficiency and defined bronchial abnormality score (generalities of the bronchial division, bronchiectasis severity, extent of bronchiectasis, peribronchial thickening, mucus plugs) identified by HRCT scan and MRI. **b** Number of patients with primary antibody deficiency and defined parenchymal abnormality score (*abscesses*, consolidations, bullae, emphysema, nodules number and dimension) identified by HRCT scan and MRI. *Gray bars* refer to MRI data; *black bars* refer to HRCT scan data

We observed high levels of concordance between HRCT and MRI in scoring parenchymal abnormalities: consolidation, *p* < 0.0001; nodule number, *p* < 0.0001; nodule dimension, *p* < 0.0001; emphysema, *p* < 0.0001). Ten out of 18 patients had at least a nodule identified by HRCT and MRI. Nodule dimension was similarly scored with exception of two patients with small nodules (diameter <1 cm) identified by HRCT scan only. Figure 1 shows the comparison between HRCT scan and MRI scores. Supplementary Table 2 shows individual scores by MRI and HRCT scan.

#### PFTs and Lung Radiological Abnormalities by MRI

**Fig. 2:**
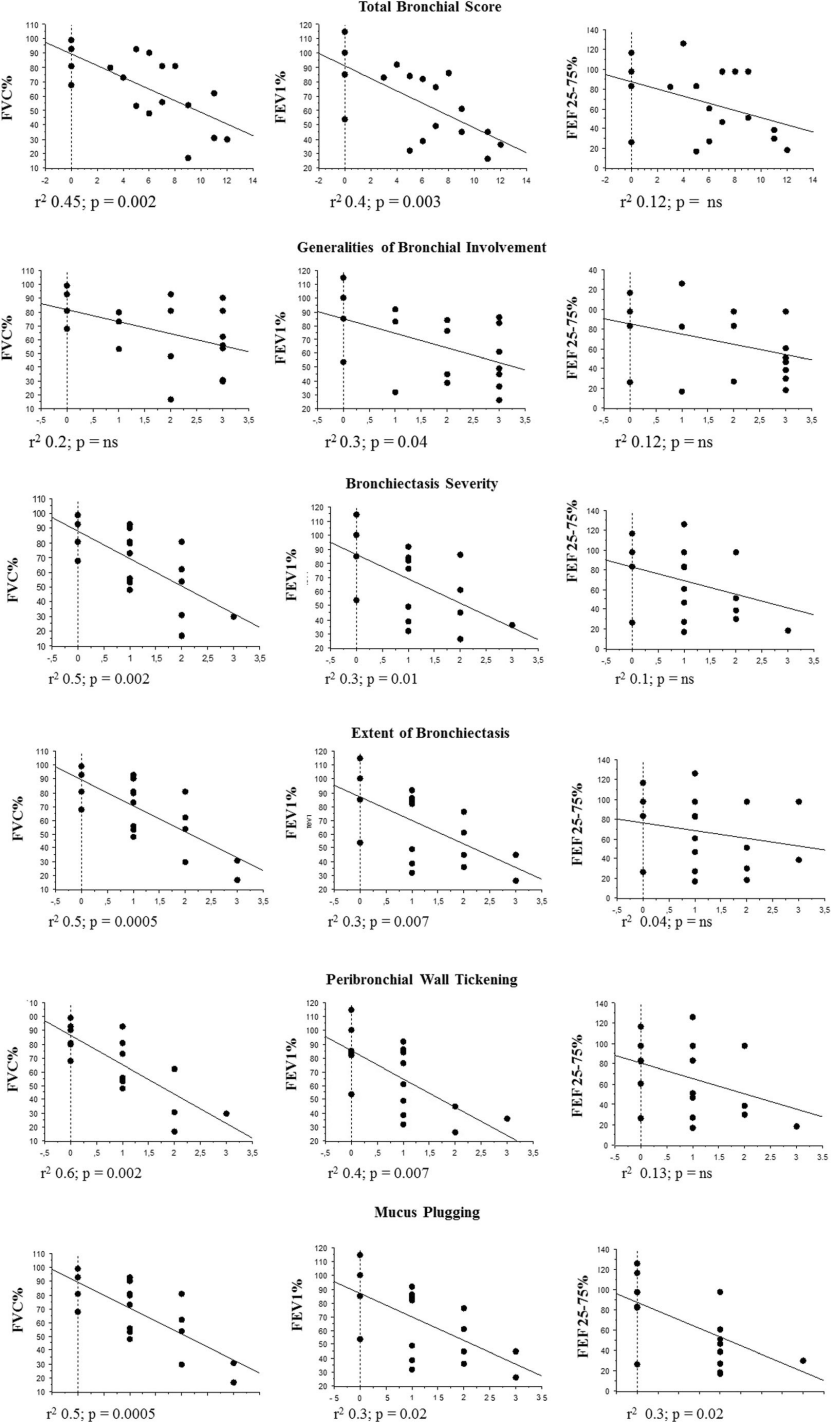
Pulmonary Function Test and Bhalla bronchial score Linear regression analysis of PFTs and bronchial parameters by MRI scored according to Bhalla; r2 and p value are indicated for each parameter

Overall, a statistically significant negative correlation was found between total bronchial scores and PFTs parameters (FEV1, *r*2 = 0.42 *p* = 0.003; FVC, *r*2 = 0.45 *p* = 0.002; PEF, *r*2 = 0.4 *p* = 0.006). In detail, FEV1 was negatively related with all bronchial parameters; FVC was negatively related with bronchiectasis severity, bronchiectasis extension, bronchial wall thickening; FEF 25–75 was negatively related with mucus plugging only (Fig. 2). Three patients (n. 4, 5, 10) with normal PFTs parameters had bronchial abnormalities (mucus plugging and bronchial wall thickening) detectable by MRI.

Total parenchymal scores did not show any correlation with PFTs, including D_LCO._

#### MRI DWI Study

**Fig. 3:**
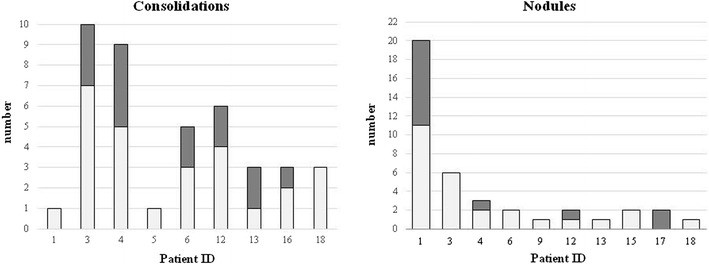
Parenchymal abnormalities with and without DWI hotspots Number of consolidations (**a**) and nodules (**b**) with or without DWI hot spots in PAD patients. *Grey box* refer to the number of lung abnormalities without DWI hotspots; *black box* refer to the number of lung alterations with DWI hotspots (y-axis). *Numbers on the x*-*axis* refer to patients identification number (ID)

**Fig. 4:**
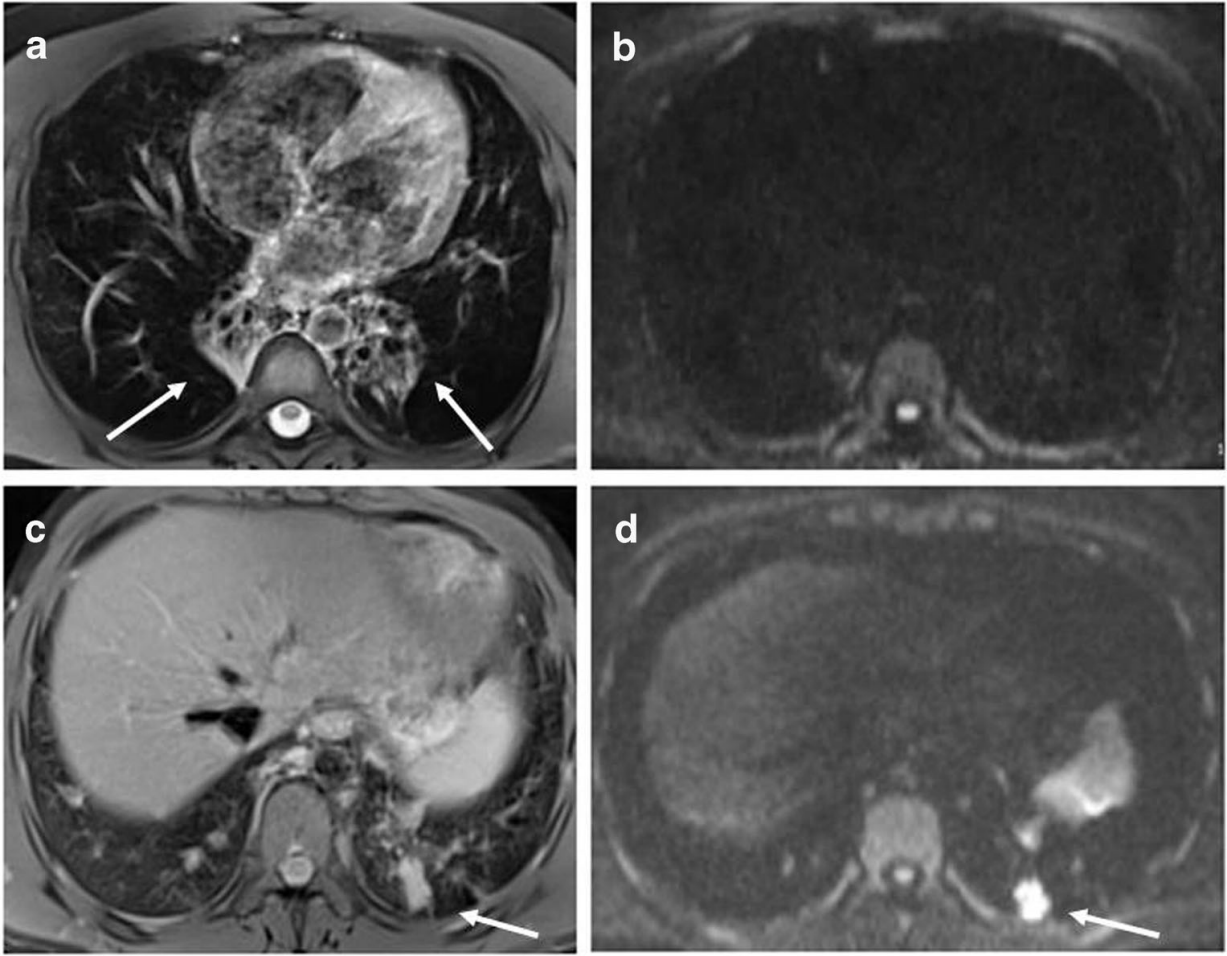
Representative MRI with DWI sequences: consolidations Consolidations detected by MRI with DWI. MRI BLADE sequence showed bilateral, chronic collapses of lower lobes (*white arrow*) (**a**) without DWI corresponding hotspots (**b**). MRI BLADE sequence showed a consolidation (*white arrow*) of the left lower lobe (**c**) with a corresponding hotspot at the DWI assessment (*white arrow*) (**d**). The histology of this consolidation by lung biopsy is shown in Supplementary Fig. 1

**Fig. 5:**
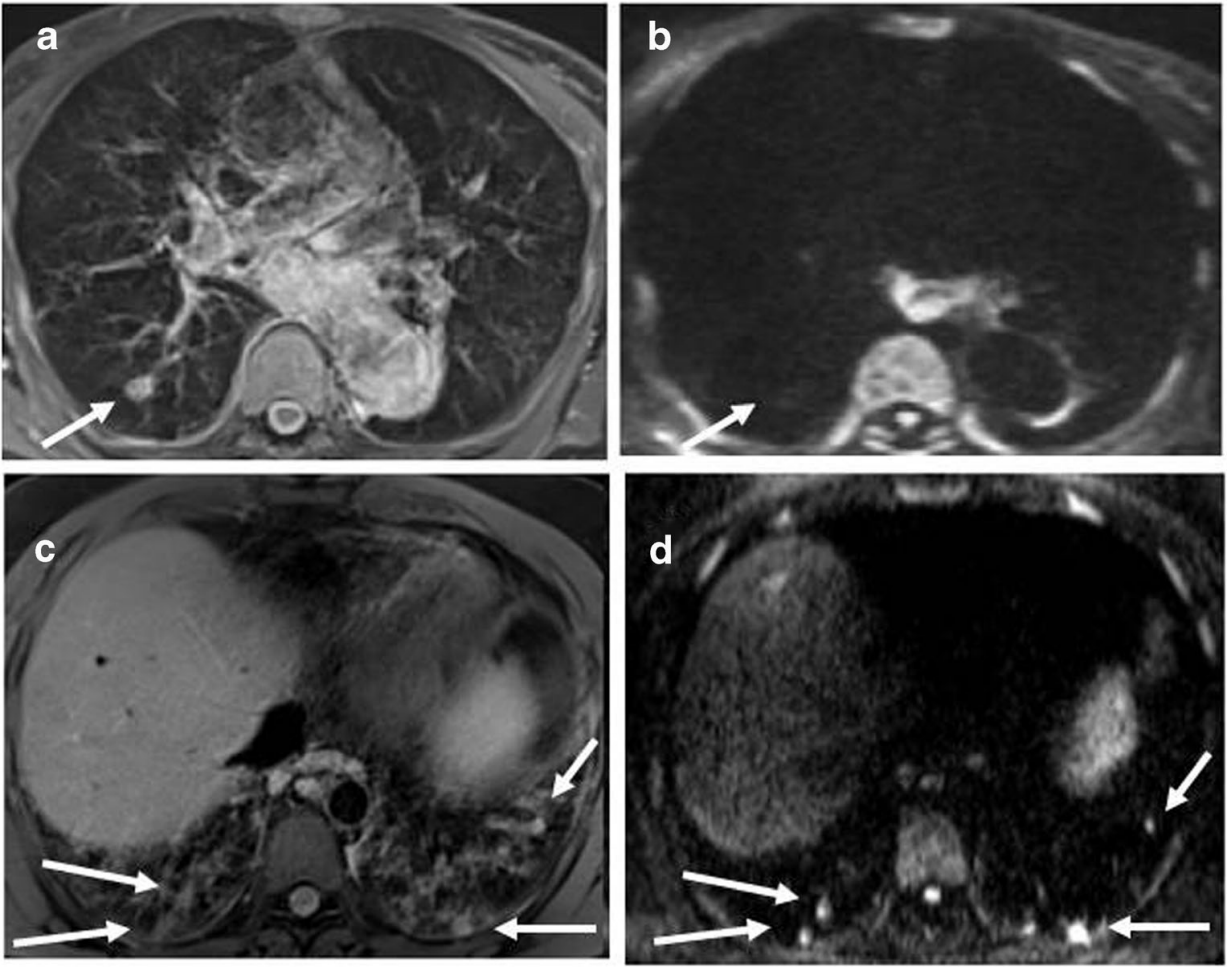
Representative MRI with DWI sequences: nodules Nodules detected by MRI with DWI. MRI BLADE sequence detecting a nodule (*white arrow*) at the right lower lobe (**a**) without a DWI corresponding hotspot (**b**). MRI BLADE sequence detecting multiple and bilateral small nodules of the lower lobes (**c**) and their corresponding hotspots at DWI assessment (*white arrow*) (**d**)

Six out of nine patients with consolidations recorded by MRI had DWI hotspots. Overall, 14 out of 41 consolidations recorded (34.1 %) were DWI restricted (Fig. 3a). DWI hotspots were related to consolidations with a MRI score <1 (segmental or lobar extension). All consolidations with a MRI score of 1 (sub-segmental extension) were DWI negative. Four out of ten patients with nodules had DWI hotspots. Overall, 13 out of 40 nodules recorded by MRI (32.5 %) were DWI-restricted (Fig. 3b). Nodules had a maximum transversal diameter ranging between 3 and 14 mm. There was no difference in the mean diameter between nodules with or without DWI hotspots (7 mm, IQR 6–8, *vs* 5.5 mm, IQR 4.75–7). In Fig. 4 and Fig. 5, we show representative MRIs with DWI-restricted and DWI-non restricted areas. Patients with DWI hotspots had higher total MRI Bhalla score and higher total MRI parenchymal scores in comparison to patients who did not have DWI-restricted parenchymal areas (total score 10 (IQR 7.5–16) *vs* 6 (IQR 2.5–8.5) (*p* = 0.05); total parenchymal score 5 (IQR 3.5–6) *vs* 0 (IQR 0–0.75), respectively). Patients with DWI-restricted consolidations had also a lower predicted FEV1% in comparison to patients without DWI hotspots (45 %, IQR 34–71 % *vs* 81 %, IQR 60.8–85 %). DWI restricted abnormalities (nodules and/or consolidations) were only detected in patients with systemic granulomatous diseases. Isolated DWI restricted consolidations were mainly present in patients with systemic lymphadenopathy (85 % *vs* 36 %, *p* = 0.04). The presence of DWI-restricted areas was not associated with splenomegaly and autoimmune manifestations. Moreover, patients with DWI-restricted abnormalities had an increased percentage of CD21^low^ B cells in comparison to patients without DWI hot spots (33 %, IQR 23.5–47, *vs* 16 %, IQR 14–21, *p* = 0.05). The analysis of other B and T cell subsets did not show any differences between patients with DWI restricted and not restricted areas.

Only one patient (n. 4) underwent a lung biopsy of in a DWI-restricted consolidation for a suspected lung lymphoma. The histological pattern showed multifocal interstitial lymphoid infiltrates spreading into the alveolar septa and surrounding airways and vessels, consistent with LIP (Supplementary Fig. 1).

#### Newly Diagnosed Patients

**Fig. 6:**
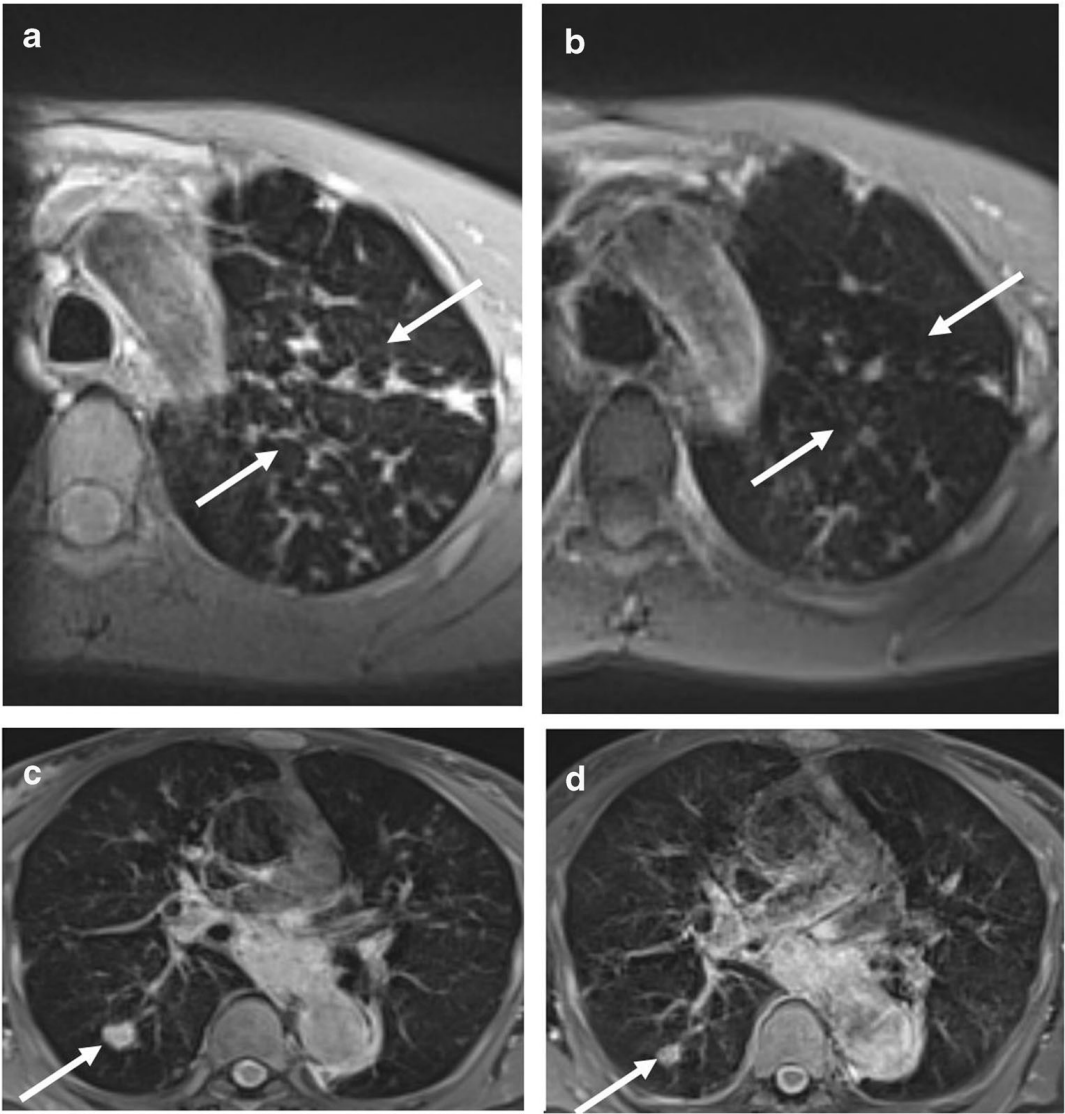
Representative MRI sequences in a patient at diagnosis and after 12 months In a 18 years old man who started Ig replacement therapy (patient n. 4), MRI BLADE sequence at first MRI showed multiple distal mucus plugs with branching patterns (“tree in bud” appearance) of the left upper lobe (*white arrows*) (**a**); these abnormalities regressed at the second MRI assessment after 12 months (*white arrows*) (**b**). MRI BLADE sequence at first MRI showed one nodule (*white arrow*) at the right lower lobe (**c**); the nodule showed a reduction in size at the second MRI assessment after 12 months (white arrow) (**d**)

Patients (n. 4, 9), who started IVIG replacement therapy at the study time, had a second lung MRI assessment and PFTs 12 months after starting Ig treatment. FEV1 improved in both patients (+8 and +7 %, respectively) as well as total parenchymal and total bronchial scores (Supplementary Fig. 2). Representative MRI sequences in a patient before and 12 months after Ig administration were shown in (Fig. 6).

## Discussion

In this study, we demonstrated that MRI with DWI identified lung inflammatory lesions in patients with PAD. Moreover, we confirmed our previous data on the non-inferiority of MRI *vs* HRCT scan [29]. HRCT is the gold standard in the lung structural damage evaluation for its excellent spatial resolution, fast acquisition times and wide availability [30]. However, HRCT poses concerns regarding the radiation dose, mostly in CVID patients, a population with a suggested increased radio sensitivity undergoing a long life follow-up [14–19]. Therefore, for these subjects, radiation-free imaging would be desirable for both follow-up and therapy monitoring. Nowadays, several strategies have been developed to overcome the inherent technical limitations of lung MRI and this imaging modality is becoming a promising tool in different diseases, including primary immune deficiencies such as ataxia-telangiectasia [31]. The implementation of DWI sequences enabled the acquisition of valuable information regarding the microstructure of tissues, allowing the identification of several pathological processes, including active inflammation [21–24, 32, 33]. In a previous cross-sectional study, we have demonstrated that MRI with BLADE sequences was a reliable technique in the detection of bronchial and parenchymal alterations and provided information overlapping HRCT [20]. We confirmed our previous data and we showed high levels of concordance between HRCT and MRI in scoring bronchiectasis severity, extent of bronchiectasis and mucus plugging. However, HRCT had a higher capacity to identify the involvement of bronchial generation up to the fifth and distal. We showed here that the inclusion of DWI sequences might provide valuable records to detect the presence of areas of active inflammation. Other radiological procedures, such as Positron Emission Tomography-CT, have been proposed for localization and quantification of active inflammation in the lung [34, 35]. However, their use is restricted by high ionizing radiation exposure, cost and limited availability.

For DWI image analysis, we used an adapted Bhalla scoring system taking into account all the alterations showing equal or higher signal intensity compared to the spinal cord at the highest b value. This technique, already adopted in previous experiences [36], did not imply a quantitative analysis derived from Apparent Diffusion Coefficient maps, it is hardly adaptable to lung imaging for its low proton density, B0 inhomogeneity, and physiologic motion. Moreover, the presence of susceptibility artefacts would lead to difficulty and poor reproducibility in selecting a consistent region of interest.

In this cohort, MRI protocol was able to identify lung alterations occurring in PAD patients; our results showed that a semi-quantitative assessment with a scoring system was feasible and matched well with the total mean HRCT scores and PFTs of the same patients. In our cohort, the MRI evaluation showed that bronchial abnormalities were the prevalent finding in PAD patients. These abnormalities might be considered as signs of chronic, active inflammation and/or infections. [1–5]. Thus, as previously suggested [3] aside from Ig replacement, a strategy to reduce the chronic mucosal damage should be defined. Chronic antibiotics treatment was suggested but its efficacy is still matter of discussion [37]; now we are conducting a large double blind study about prophylaxis with azithromycin in PAD in order to prove a possible benefit in reducing respiratory exacerbations as shown in patients with non-cystic fibrosis bronchiectasis [38]. Even if Ig therapy could not reduce the progression of lung diseases and damage, we documented an improvement of parenchymal and bronchial scores and of PFTs in newly diagnosed PAD patients after the beginning of Ig replacement therapy. We found a significant relationship between the extent of MRI bronchial abnormalities score and PFTs. Based on these findings, it appears that PFTs alterations were strictly connected to a bronchial damage. However, MRI parenchymal scores did not correlate with PTFs, including D_LCO._ This was not surprising since none of the patients enrolled had bullae, only few patients had a mild emphysema and none had clinical signs of acute pulmonary infection at the time of the study. Moreover, MRI was able to identify active bronchial abnormalities in a group of patients with normal PFTs. The interpretation of DWI findings may represent a useful tool in addition to the conventional morphological analysis, mostly in subjects with advanced disease. In our evaluation, all DWI hotspots corresponded to morphological alterations, but not vice versa: lung pathological findings showed different DWI patterns or no DWI signal at all. It can be postulated that the mismatch between DWI and morphological data correlates with cellular and extra-cellular space modifications (density/composition/water content) induced by active inflammation: these phenomena may affect DWI even before macroscopic alterations can be detected on conventional imaging. DWI parenchymal restricted areas were mainly detected in patients with severe total and parenchymal scores and with more severe alterations of PFTs. They were associated with abnormalities described in patients with the inflammatory CVID phenotypes: systemic granulomatous disease, systemic lymphadenopathy, expansion of CD21^low^ B cells [39]. For these reasons, DWI might represent a marker of disease activity and it could be used during follow-up of PAD patients in order to distinguish *foci* of active inflammation/sub-clinically progressive lung disease from chronic pathological manifestations [8–11]. The presence of inflammation represents an important pathological process underlying many lung morphological alterations in PAD patients and, in the clinical practice, it is important to determine the activity of disease to guide interventions. DWI sequences, with the possible identification of active inflammation, may allow an early discrimination of patients with infective/inflammatory or non-infective/inflammatory disease. Inflammatory lung diseases in CVID have been reported to be associated with lung disease progression and increased mortality [11, 40, 41], but the timing when starting an immunosuppressive treatment is still open [42–44]. Moreover, since MRI is a radiation-free technique, it can be repeatedly used in follow-up monitoring of treatment response, differentiating between active and fibrotic lesions.

## Conclusions

Clinicians might consider MRI with DWI in the diagnostic algorithm of pulmonary lesions as a non-invasive, radiation-free method complementary to other diagnostic tools. Our results might help to define the role of morphological MRI sequences and DWI sequences in the diagnosis of *foci* of active inflammation and/or sub-clinically progressive lung disease. The major limit of this study was the lack of information on bronchoalveolar lavages and the presence of a single DWI restricted lung biopsy. Future studies could likely benefit from the longitudinal evaluation of larger cohorts of patients with primary immune deficiencies or other clinical conditions requiring a frequent ionizing radiation exposure in order to assess the potential use of DWI to select biopsy areas and to monitor the response to treatment in non-infective inflammatory disease.

## Electronic supplementary material

Supplementary Figure 1 Histological pattern by lung biopsy. Lung biopsy showed multifocal interstitial lymphoid infiltrates (white arrows) spreading into the alveolar septa and surrounding airways and vessels (a). The infiltrates consisted of polyclonal B cells (CD20+) mainly organized in nodules (b). As already described in CVID, the LIP infiltrate did not show a Bcl2 positive mantle zone (c) surrounding a Bcl2 negative/Bcl6 positive germinal center (c–d). CD4+ T cells were the prevalent T cell subset located among B cells agglomerates, while CD8+ T cells were rarely observed (e–f). The histological pattern was consistent with the diagnosis of lymphocytic interstitial pneumonia (LIP). (a) hematoxylin and eosin stain, original magnification 10×; (b) CD20 immunostaining, original magnification 20×; (c) Bcl2 immunostaining, original magnification 20×; (d) Bcl6 immunostaining, original magnification 20x; (e) CD4 immunostaining, original magnification 20×); (f) CD8 immunostaining, original magnification 40 × . (JPEG 228 kb)

Supplementary Figure 2 Score of individual lung abnormalities in newly diagnosed patients at diagnosis and after 12 months. Total Bronchial (a) and Total Parenchymal Scores (b) in the two patients (n.4 and 9) who started Ig replacement therapy at the time of the study. White bars refer to first MRI assessment; gray bars refer to second MRI assessment. Patient n.4 showed an improvement on the bronchial generation involved, mucus plugging, nodules number and dimension and on consolidation. Patient n.9 showed the improvement on nodules number and dimension. (JPEG 51 kb)

Supplementary Table 1 Diagnosis, clinical features and FEV1 of 18 patients enrolled in the study (PDF 36 kb)

Supplementary Table 2 Comparison of individual scores by MRI and HRCT Individual bronchial and parenchymal abnormalities by MRI and HRCT according to a modified Bhalla score in 18 PAD patients. (PDF 29 kb)
