# Supplementary figures and images for: Lung Magnetic Resonance Imaging with Diffusion Weighted Imaging Provides Regional Structural as well as Functional Information Without Radiation Exposure in Primary Antibody Deficiencies
Source: J Clin Immunol. 2015 Jun 12;35(5):491–500. doi: 10.1007/s10875-015-0172-2 (PMC4502290; doi:10.1007/s10875-015-0172-2)

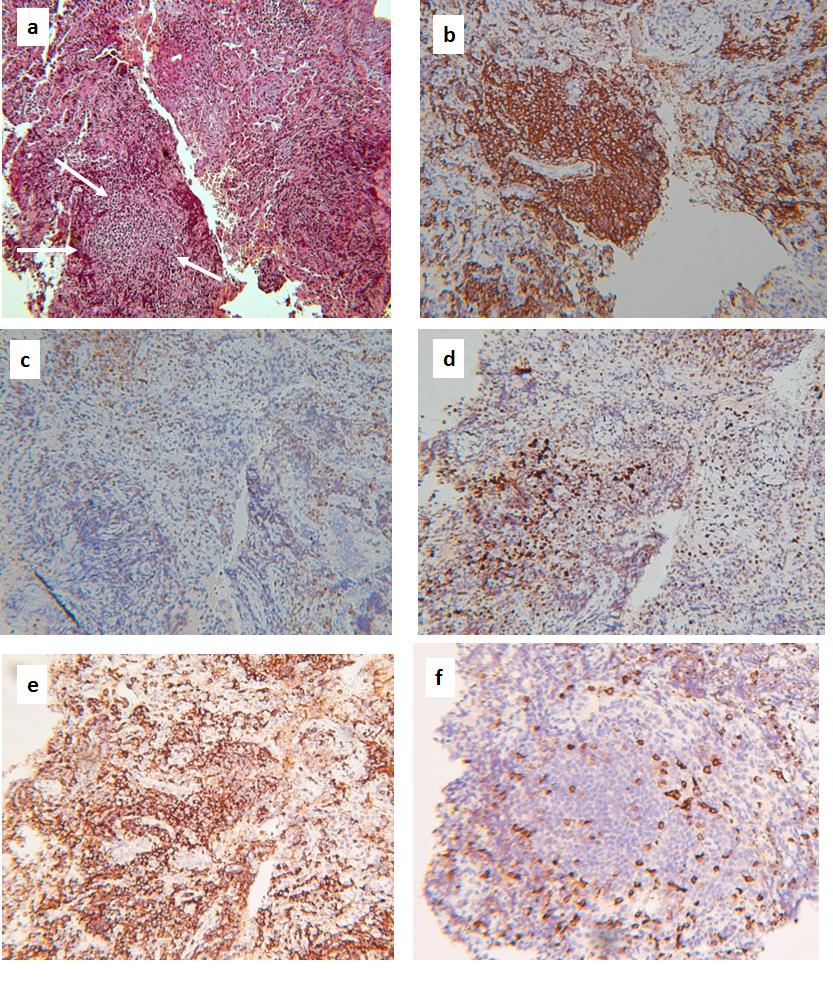

Supplement: Supplementary file 1 — Histological pattern by lung biopsy. Lung biopsy showed multifocal interstitial lymphoid infiltrates (white arrows) spreading into the alveolar septa and surrounding airways and vessels (a). The infiltrates consisted of polyclonal B cells (CD20+) mainly organized in nodules (b). As already described in CVID, the LIP infiltrate did not show a Bcl2 positive mantle zone (c) surrounding a Bcl2 negative/Bcl6 positive germinal center (c–d). CD4+ T cells were the prevalent T cell subset located among B cells agglomerates, while CD8+ T cells were rarely observed (e–f). The histological pattern was consistent with the diagnosis of lymphocytic interstitial pneumonia (LIP). (a) hematoxylin and eosin stain, original magnification 10×; (b) CD20 immunostaining, original magnification 20×; (c) Bcl2 immunostaining, original magnification 20×; (d) Bcl6 immunostaining, original magnification 20x; (e) CD4 immunostaining, original magnification 20×); (f) CD8 immunostaining, original magnification 40 × . (JPEG 228 kb) [file 10875_2015_172_Fig7_ESM.jpg]

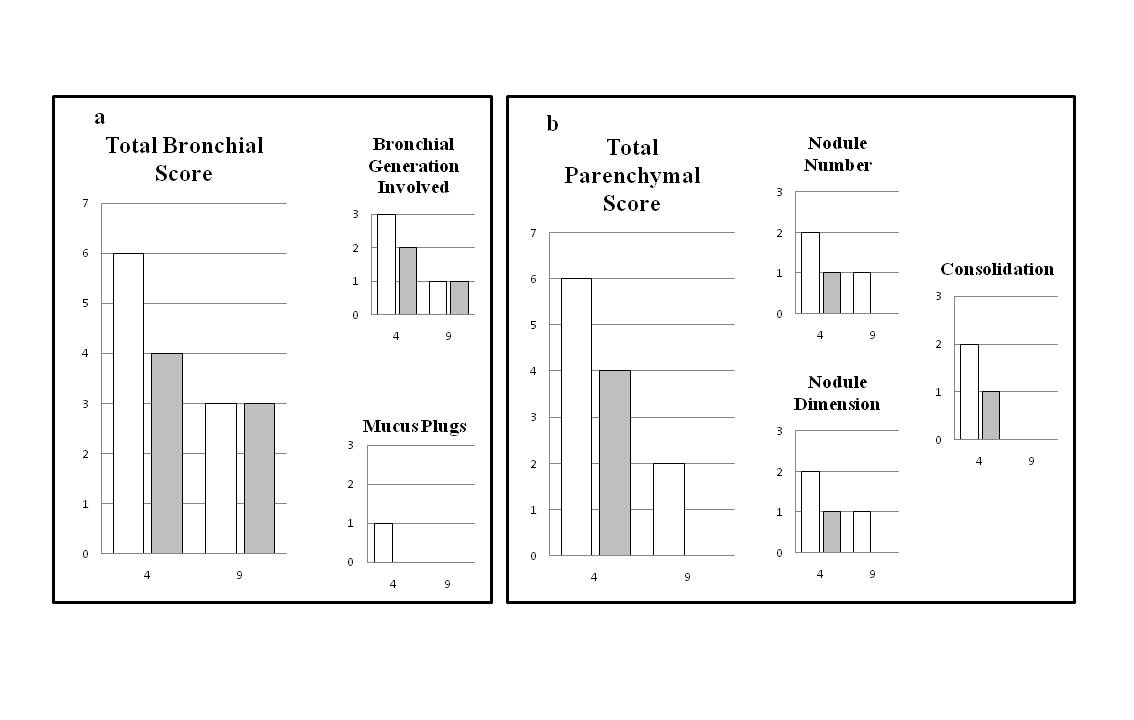

Supplement: Supplementary file 2 — Score of individual lung abnormalities in newly diagnosed patients at diagnosis and after 12 months. Total Bronchial (a) and Total Parenchymal Scores (b) in the two patients (n.4 and 9) who started Ig replacement therapy at the time of the study. White bars refer to first MRI assessment; gray bars refer to second MRI assessment. Patient n.4 showed an improvement on the bronchial generation involved, mucus plugging, nodules number and dimension and on consolidation. Patient n.9 showed the improvement on nodules number and dimension. (JPEG 51 kb) [file 10875_2015_172_Fig8_ESM.jpg]
