# Supplementary material for: Lung Magnetic Resonance Imaging with Diffusion Weighted Imaging Provides Regional Structural as well as Functional Information Without Radiation Exposure in Primary Antibody Deficiencies
Source: J Clin Immunol. 2015 Jun 12;35(5):491–500. doi: 10.1007/s10875-015-0172-2 (PMC4502290; doi:10.1007/s10875-015-0172-2)
Supplement: Supplementary file 3 — Diagnosis, clinical features and FEV1 of 18 patients enrolled in the study (PDF 36 kb) [file 10875_2015_172_MOESM1_ESM.pdf]

supplementary tables
Click here to download Electronic Supplemental Material (ESM): tables.docx

Supplementary Table 1 – Diagnosis, clinical features and FEV1 of 18 patients enrolled in the study

Patient Number	Diagnosis	Gender	Age (years)	Disease time (years)	Trough levels	IVIG/SCIG replacement	Splenomegaly	Systemic granulomatous disease	Autoimmunity	Linfo-adenopaty	FEV1%	COPD
1	CVID	M	36	4	645	IVIG	Yes	Yes	Immune Thrombo-cytopenic Purp ura; psoriasis	Yes	76	No
2	CVID	M	43	13	958	IVIG	Yes	No	no	Yes	61	Yes
3	CVID	F	39	6	601	IVIG	Yes	Yes	Haemoltytic anemia, diabetes mellitus	Yes	92	No
4	CVID	M	18	1	650	IVIG	No	Yes	Lymphocytic interstitial pneumonia	Yes	82	No
5	CVID	M	40	22	613	IVIG	Yes	No	no	No	86	No
6	CVID	M	47	25	668	IVIG	Yes	Yes	haemolitic anemia	Yes	45	no
7	CVID	M	48	2	550	IVIG	Yes	No	no	Yes	49	yes
8	CVID	M	30	10	509	SCIG	Yes	No	Immune Thrombocytop enic Purpura	No	39	No
9	CVID	F	24	2	528	SCIG	No	No	Celiac disease	No	83	No
10	CVID	M	55	44	704	IVIG	No	No	no	No	84	No
11	CVID	M	27	2	850	IVIG	Yes	No	no	No	85	No
12	CVID	F	64	56	603	IVIG	Yes	No	no	Yes	26	Yes
13	XLA	M	35	33	950	IVIG	Yes	No	no	No	36	Yes
14	XLA	M	24	12	551	IVIG	No	No	Celiac Disease, Ulcerative Rettocolitis, Alopecia	Yes	45	Yes
15	CVID	F	64	15	757	IVIG	No	No	Arthritis	No	115	No
16	CVID	M	32	3	500	IVIG	No	No	no	Yes	32	Yes
17	CVID	M	70	8	836	IVIG	No	No	no	No	100	No
18	CVID	F	73	8	725	IVIG	Yes	No	no	Yes	54	Yes
