# Supplementary material for: Lung Magnetic Resonance Imaging with Diffusion Weighted Imaging Provides Regional Structural as well as Functional Information Without Radiation Exposure in Primary Antibody Deficiencies
Source: J Clin Immunol. 2015 Jun 12;35(5):491–500. doi: 10.1007/s10875-015-0172-2 (PMC4502290; doi:10.1007/s10875-015-0172-2)
Supplement: Supplementary file 4 — Comparison of individual scores by MRI and HRCT Individual bronchial and parenchymal abnormalities by MRI and HRCT according to a modified Bhalla score in 18 PAD patients. (PDF 29 kb) [file 10875_2015_172_MOESM2_ESM.pdf]

Supplementary Table 2. – Comparison of individual scores by MRI and HRCT

Individual bronchial and parenchymal abnormalities by MRI and HRCT according to a modified Bhalla score in 18 PAD patients.

PATIENT NUMBER	GENERALITIES OF THE BRONCHIAL DIVISION		BRONCHIECTASIS SEVERITY		BRONCHIECTASIS EXTENSION		BRONCHIAL WALL THICKENING		MUCUS PLUGGING		ABSCESSES		BULLAE		EMPHYSEMA		NODULE NUMBER		NODULE DIMENSION		CONSOLIDATION	
	CT	MRI	CT	MRI	CT	MRI	CT	MRI	CT	MRI	CT	MRI	CT	MRI	CT	MRI	CT	MRI	CT	MRI	CT	MRI
**1**	3	2	1	1	2	2	1	1	1	1	0	0	0	0	0	0	2	2	2	2	1	1
**2**	3	3	2	2	2	2	2	1	1	1	0	0	0	0	0	0	0	0	0	0	0	0
**3**	2	1	1	1	1	1	1	1	0	0	0	0	0	0	0	0	2	2	2	2	2	2
**4**	3	3	1	1	1	1	1	0	1	1	0	0	0	0	0	0	2	2	2	2	2	2
**5**	3	3	2	2	1	1	1	1	1	1	0	0	0	0	0	0	0	0	0	0	1	1
**6**	2	2	2	2	3	3	2	2	0	0	0	0	0	0	1	1	1	1	2	2	2	2
**7**	3	3	1	1	1	1	1	1	1	1	0	0	0	0	0	0	0	0	0	0	0	0
**8**	3	2	1	1	1	1	1	1	1	1	0	0	0	0	0	0	0	0	0	0	0	0
**9**	3	1	1	1	1	1	0	0	0	0	0	0	0	0	0	0	1	1	1	1	0	0
**10**	2	2	1	1	1	1	1	1	0	0	0	0	0	0	0	0	0	0	0	0	0	0
**11**	1	0	0	0	0	0	0	0	0	0	0	0	0	0	0	0	0	0	0	0	0	0
**12**	3	3	2	2	3	3	2	2	1	1	0	0	0	0	2	2	1	1	1	1	2	2
**13**	3	3	3	3	2	2	3	3	1	1	0	0	0	0	2	2	1	1	1	1	2	2
**14**	3	3	2	2	2	2	2	2	2	2	0	0	0	0	0	0	1	0	1	0	0	0
**15**	0	0	0	0	0	0	0	0	0	0	0	0	0	0	0	0	1	1	1	1	0	0
**16**	2	1	1	1	1	1	1	1	1	1	0	0	0	0	0	0	1	0	1	0	2	2
**17**	0	0	0	0	0	0	0	0	0	0	0	0	0	0	0	0	1	1	1	1	0	0
**18**	3	0	1	0	1	0	1	0	0	0	0	0	0	0	1	0	1	1	2	2	1	1
